# Towards validating invalidated knowledge: a discourse analysis of firsthand accounts of hearing voices

**DOI:** 10.1186/s40359-024-02023-x

**Published:** 2024-10-02

**Authors:** Lill Susann Ynnesdal Haugen

**Affiliations:** Department of Health Promotion and Development, The Faculty of Psychology, University of Bergen, Bergen, Norway

**Keywords:** Hearing voices, Voice-hearing, Auditory hallucinations, Foucauldian discourse analysis, Firsthand experiences, Firsthand knowledge, Biomedical discourse, Epistemic violence, Hearing Voices Movement (HVM)

## Abstract

**Background:**

As Foucault historically traced, dialogs about madness were silenced with the emergence of biomedical psychiatry. The silence entailed the epistemic violence of invalidating persons who hear voices as knowers, arguably leaving them without validating sensemaking languages for firsthand experiences. This article analyzes five Norwegian firsthand accounts of hearing voices, and how they differed from the predominating biomedical psychiatry discourse, in search of validating languages and knowledge that may facilitate making sense of voice-hearing for persons who hear voices.

**Methods:**

The text material consisted of four sets of blogs authored by four young women and a short interview with a man, all of whom had firsthand experiences of voice-hearing in a Norwegian context. Ian Parker’s version of Foucauldian discourse analysis was used to analyze the material.

**Results:**

Six discourses were identified: *biomedical psychiatry-discourse*,* discourse of reason*,* psychodynamic discourse*,* discourse of personal characteristics*,* spiritual discourse and discourse of personal relationships between hearer and voices*. Within the discourses of biomedical psychiatry and reason, voice-hearing was rendered as hallucinations, unreason, and as a problem to be solved, preferably by professionals, thus silencing the person who hears voices. In contrast, within the discourses of psychodynamics, personal characteristics, spirituality, and personal relationships between hearer and voices, voice-hearing has diverse meanings, and they grant voice hearers greater freedom concerning voice-hearing. The psychodynamic discourse nonetheless aligns with the discourses of reason and biomedical psychiatry in allowing professionals the prerogative of determining the meaning of voice-hearing. The remaining three discourses appear to facilitate more space for voice-hearers to narrate and engage with their voice-hearing at their discretion, with little to no professional impingement.

**Conclusions:**

Discursive complexity notwithstanding, I consider that particularly the discourses of personal characteristics, spirituality, and personal relationships facilitate languages that may enable a person to narrate their own experiences and actions at their own discretion, without needing an expert commentator on the side. To have such languages available is argued to entail clear strides towards more empowered positions in one’s life.

## Background

In a recently published handbook of Mad Studies, *ex-mental health service user/survivor and activist* Mary O’Hagen [1] reflects on her four decades of involvement in the *International mental health service users’/survivors’ movement* (from hereon ‘service user’s movement’). In dialog with Peter Beresford, O’Hagen contends that, despite years of advocacy for change on behalf of people in psychosocial hardships, the biomedical psychiatry-discourse still predominates. In her documentary film, *The Politics of Memory*, survivor, activist, and psychologist Patricia Deegan [2] has succinctly articulated that the master narrative of biomedicine pushes other ways of knowing and living with *madness* to the ‘fringes of public perception.’ O’Hagen [1] argues that without actively working on structural and systemic changes to dethrone biomedical psychiatry from its hegemonic position, strides towards social justice for *Mad People* as a social group will remain negligible. Looking to Mad Studies and its proponents, such as O’Hagen, there are calls for generating and sharing research-based knowledge about firsthand accounts of psychosocial differences and hardships and, in turn, validating firsthand knowledge [3]. As a scholar-practitioner from critical community psychology, I align with these calls from Mad Studies and seek to contribute to validating firsthand knowledge about hearing voices through this article. Generating and sharing research-based knowledge about firsthand accounts are among the avenues that can help build a critical mass of support that actively promotes change. Contributing to this is also an aim of the article.

### Silencing and invalidating madness

In *Madness and Civilization* (1988/1961), Michel Foucault [4] historically analyzed discourses of madness from the Middle Ages through the Age of Enlightenment. He traced that dialogs about madness were silenced with the emergence of discourses of reason and psychiatry. These discourses colonized madness, effectively silencing the mad person by stripping away a validating language to put what was seen, heard, and felt into meaning.

In contemporary societies where the Western biomedical model of psychiatry is prevalent, hearing voices and other sounds, seeing visions, and smelling odors that others cannot are usually considered to be among the most prominent signifiers of madness. Within the biomedical psychiatry-discourse, such experiences are categorized as symptoms of psychiatric diagnoses, particularly psychosis [5]. In the biomedical psychiatry discourse, the symptom of hearing voices is considered devoid of meaning, much as frequent urination is considered a symptom of diabetes [6]. Without meaning, there cannot be a validating language from which to articulate firsthand experiences.

Despite being more common than is generally acknowledged, with around 4–10% of the general population in various countries reporting experiences of hearing voices that others do not [7–10], hearing voices represents a unique firsthand experience and knowledge that has historically been silenced and invalidated. In this article, I have chosen to address this non-dominant and diverse social group as *people who hear voices*. In the biomedical psychiatry-discourse, an aim for the ‘treatment’ of the ‘psychiatric patient’ who hears voices is to gain ‘insight.’ *Insight* involves coming to share the belief that hearing voices is simply an empty symptom, and consequently being compelled to ignore, deny, and not accept one’s firsthand experiences. However, even within psychiatric research, it is documented that insight is, unsurprisingly, associated with the added burden of experiencing increased stigma, and reduced hope and quality of life [11]. However, biomedical psychiatry’s perspective on voice-hearing is being challenged by persons who hear voices and their allies, in particular through the branch of the service users’ movement called the *Hearing Voices Movement* (HVM).

### Hearing voices, resistance, and stigma

The HVM was established in the 1980s in the Netherlands, led by the psychiatrist Romme, his patient Hage, and his wife and journalist Escher [6, 12, 13]. In the HVM, voice-hearing is understood as a normal variation in the complexities and range of human experiences rather than being reduced to a symptom of psychosis and mental disease [12].

In support of the claim of voice-hearing being a variation in the complexities and range of human experiences, Johns et al. [8] estimated that only 25% of the British voice-hearing population qualified for a diagnosis relating to psychosis. In their systematic review of the incidence and prevalence of subclinical hallucinations, van Os et al. [10] reported that only a few subclinical individuals could be considered at risk of ever qualifying for a psychiatric diagnosis. As such, following the HVM and mainstream epidemiology, hearing voices cannot be reduced solely to a symptom of serious psychiatric diagnosis. Thus, other ways of experiencing, articulating, and knowing voices need to be explored in their own right, as is the aim of this article.

In countries such as the United Kingdom and the Netherlands, the HVM is considered to have made strides to promote that professionals are learning from people who hear voices [14]. From when I first began work on this project in the early 2010s until the present day in 2024, these strides also appear to be reflected in a growing literature that illuminates firsthand accounts of hearing voices. In this literature, firsthand knowers have discussed their voice-hearing through diverse lenses – for instance, as biomedical symptoms and psychiatric diagnoses [15, 16], psychological phenomena and activity [15, 17], traumas [6, 14, 18], the spiritual sphere [17, 19], and personas, relationships and identity [16, 20, 21].

Important progress notwithstanding, as discussed by O’Hagen [1], most people who speak of voices – especially in relation to being in distress – are still typically subjected to the hegemony of biomedical psychiatry, both in the mental health system and civil society. In many cases, they do not know about and are not getting to know about alternative ways of being in the world and seeing the world with voices. As discussed in the editorial of the current BMC Psychology-collection on *Mental Health*,* Discourse*,* and Stigma*, by Zayts-Spence, Edmonds, and Fortune [22], there are strongly discrediting micro- to macro-sociological workings of stigma related to people in psychosocial distress. Inquiries have related hearing voices to the person being stigmatized and invalidated both in the gaze of civil society [16] and in the gaze of oneself [23].

In Mad Studies, scholars are concerned with inquiring into the particular systematic stigmatization and exclusion of people with psychosocial differences, referred to as sanism [24]. *Sanism* concerns the notion that it is normal and right to be whatever is considered sane (at the time and place) and that it is pathological and wrong to be whatever is considered psychosocially different [24, 25]. Sanism resembles other concepts that address the systematic underprivileging of various groups, such as racism, sexism, and ableism. Sanism has even been found in otherwise typically progressive contexts, such as academia [24].

In the firsthand account by voice-hearer and doctor in psychology, Eleanor Longden [18], she discusses being met with invalidation by the biomedically predominated psychiatric system, and with stigmatization and othering in civil society, in what she considers as a period in life where she was deeply insecure and instead in dire need of being validated and listened to as a human being. Longden and others strongly [3] criticize the standard modus operandi of the mental health system for doing more harm than good when meeting new persons who are in a life crisis and are hearing voices. Within Mad Studies, the postcolonial concept of *epistemic violence* addresses the structural and systemic invalidation of the firsthand experiences and knowledge of people in psychosocial hardships [26], as exemplified by Longden’s account.

The importance of promoting the availability of different ways of seeing, living with, and articulating voices that make personal meaning for persons who hear voices is emphasized both in personal accounts such as the one above and in scholarly inquiries (e.g., 6, 21), and by the fact that predominating discourses have a history of repeatedly invalidating alternative knowledge related to psychosocial differences [25]. The following quote by O’Hagen [27, para. 8] paints a poetic metaphor that illuminates the magnitude of the epistemic violence that people with psychosocial differences have historically been subjected to: “Our madness stands outside in the dark, knocking on the door to meaning, struggling to get in.”

### Theoretical and methodological framework

This inquiry’s theoretical and methodological framework is Foucauldian discourse analysis in psychology, as formulated by Ian Parker [28]. Drawing on Foucault, Parker’s (p. 5) working definition of discourse is “a system of statements which constructs an object” [28]. The definition is further conditioned on discourses being: coherent systems of meaning constructing particular objects and subjects; situated in culture and history; realized in texts; and reflective on themselves and other discourses. Additionally, discourses are argued to support societal institutions, reproducing relations of power and having ideological effects [28].

Foucauldian discourse analysis enables an exploration of firsthand knowledge about hearing voices in a respectful and ethically reflexive way [13, 29]. Furthermore, it facilitates critical investigations of predominating psychiatric and psychological (‘psy’) discourses and their privileging of some forms of human experiences and actions and the derogation of others [13, 29]. Working on destabilizing predominating psy-discourses and facilitating space for resistance and increased freedom of movement for those underprivileged by them are reasons for conducting discourse analysis [28].

This article analyzes five Norwegian firsthand accounts of hearing voices, and how they differ from the predominating biomedical psychiatry discourse, in search of validating languages and knowledge that may facilitate sensemaking of voice-hearing for persons who hear voices. Furthermore, through this article, I aim to share research based-knowledge concerning firsthand accounts of hearing voices in order to help build a critical mass of support that actively promotes change for people with psychosocial differences. The following research questions guided the inquiry: How do five Norwegian firsthand knowers of hearing voices discuss voice-hearing, and may some of the discourses reconstructed from the discussions constitute discursive alternatives to the predominating biomedical psychiatry discourse?

## Methods

### Blogs

The inquiry started as a master’s thesis project in psychology, which was submitted in the Norwegian language in 2011. The main text material of the inquiry was firsthand information about voice-hearing from already written posts in Norwegian open-access blogs. At the end of November 2010, I emailed six Norwegian blog authors, informed them about the inquiry, and asked for permission to use posts regarding voice-hearing from their blogs from circa October 2009 to November 2010 for my research. Four Norwegian blog authors answered and consented to participate in the inquiry by allowing me to use the content in their blogs for qualitative research- and educational purposes. Those who wanted a copy of the finished master’s thesis received it. All were young women in their twenties. They were given the pseudonyms ‘Amalie,’ ‘Liv,’ ‘Nadia,’ and ‘Sofie.’

Throughout this paper, concern for the bloggers’ anonymity and integrity outweighed the presentation of information about the blogs or the bloggers that could possibly identify them via Internet searches. Therefore, only general descriptions of the bloggers and how the blogs were selected are provided. Some blogs were found through general Internet searches, with Norwegian search terms related to hearing voices and blogging. These blogs linked to other blogs related to hearing voices, some of which were also included. Inclusion criteria were: (i) clearly stated personal experiences of voice-hearing and (ii) an ample amount of blog posts concerning voice-hearing from an experience-based perspective. The process of finding and selecting blog posts from the four blogs for close analysis included using search terms such as ‘voice-hearing’ and, where applicable, the names of voices in integrated search functions in the blogs and via advanced Google searches. Blog posts were evaluated and included according to the extent to which (a) a post contained explicit references to voice-hearing, (b) a post added to the diversity of information about voice-hearing, and (c) there was an adequate amount of dedicated text about hearing voices. Selected texts were gathered in a Word document for each blogger, stripped of identifying information, and securely stored to protect the bloggers’ anonymity.

In the *Results and Discussion* section, data material from Liv’s blog is presented as verbatim quotes, while the blogs of Amalie, Nadia, and Sofie are presented solely in paraphrased form to promote their anonymity and integrity. Liv has closed the blog, and it does not appear in Internet searches with selected, brief quotes in the original Norwegian language. Thus, Liv’s anonymity and integrity continue to be protected as I have chosen to include selected anonymized quotes to contribute to presenting an accountable analysis.

All quoted and paraphrased data material has been translated from Norwegian into English. The paraphrases are formulated with original words and as close to the original meaning-content as possible, often in a more condensed, slightly more general or otherwise slightly rewritten form, all to prevent identification. For instance, instead of writing the exact numbers and words referred to in Nadia’s blog about hearing voices-statistics, I paraphrase the content more generally (“a majority of those who hear voices are able to manage their voices”).

### Short interview

A short interview was conducted with a middle-aged man who had voice-hearing experiences. He contacted us after reading an information letter about the study, which was posted on bulletin boards in local NGOs for persons with a history of mental health issues. The volunteer participant was informed about the inquiry and freely and explicitly consented to participate.

The man was assigned the pseudonym ‘Thomas’ and was interviewed in 2010 by me at a university office. Thomas was asked to talk about his experiences of voice-hearing. After about five minutes, he ended the interview. Thomas did not revoke his consent, rather he expressed being satisfied with what he had said. Before leaving, he was encouraged to contact me if the participation caused him any distress. As part of the study’s ethical plan, a specialist in clinical psychology had agreed to meet distressed informants. The audiotape-recorded interview was transcribed verbatim.

The Norwegian Regional Committees for Medical and Health Research Ethics (REK), Western region, granted ethical approval to conduct the interview (reference “2010/1101”). In addition, I adhered closely to guidelines from the Norwegian National Committee for Research Ethics in the Social Sciences and the Humanities. In 2010 in Norway, to my understanding, openly published information on the Internet was considered publicly available for research purposes. To my knowledge, regulations and a research ethics authority (‘internal review boards’) were yet to be developed with regard to applying for ethical approval to engage in research on openly published information. Nevertheless, the guidelines advised that authors of information on the Internet should be asked for permission to use their material and to protect their anonymity.

### Discourse analysis

Parker’s [28] discourse dynamics facilitate engagement in discourse analyses that are concerned with moral and socio-political matters. In particular, this ‘facilitates an analysis of the functions, limits, and possibilities of discourses for the real lives of people, concerning both meaning and the material world [34, p. 49].’

Parker’s [28] criteria were used to identify and analyze discourses. I list the criteria with the same or similar words as used by Parker, and in the same order (p.3–13): a discourse (1) is realized in text; (2) is about objects; (3) contains subjects; (4) is a coherent system of meaning; (5) refers to other discourses; (6) is self-reflecting; (7) is situated in culture and history; (8) supports institutions; (9) reproduces relations of power; and (10) has ideological effects.

In the early 2010s, I was a novice in engaging practically with the craft of discourse analysis. I experienced that Parker’s discourse dynamics theoretically resonated with me, but I needed to learn about conducting discourse analysis in practice from different ‘teachers.’ Willig’s [30] stage-based approach to discourse analysis was used to support the analysis-process at that time due to the value of her pedagogical guidance and worked-out examples for an analyst-in-training. Willig’s guidance for Stage 1 discursive constructions was used together with Parker’s corresponding guidance related to criterion (2) discursive objects, which involves identifying and analyzing the main discursive objects of the inquiry, which here is the different constructions of ‘hearing voices.’ Willig’s guidance for Stage 2 discourses was used together with Parker’s guidance related to (2) discursive objects and (4) coherent system of meaning, which involves analyzing the discourses in which the relevant discursive objects are traced. Willig’s guidance for Stage 4 positionings and Stage 5 practice was used together with Parker’s guidance related to (3) subjects, which involves identifying and analyzing the subject positions of the identified discourses, and tracing the discourses’ functions, limits, and possibilities for the real lives of people. A subject position may concern a social category and its position within a given discourse – for instance, a doctor, nurse, or patient, in a biomedical discourse. How a person is positioned resonates so strongly with the person that they often ‘accept the positioning as a part of themself, whether it is privileged or underprivileged [34, p. 16].’ All subject positions involve ‘a range of possibilities, limitations, rights, and duties regulating what the person can experience, say, and do in a given time and space [34, p. 16].’

The discourse analysis was conducted with the original blog material, as authored in Norwegian by the blog authors, and with the short interview. The blog material and the short interview were first read closely and repeatedly. Next, I critically distanced myself from the texts and engaged in free associations to the literal content – preliminary tracing connections between the material’s micro-level instances of discourses to broader discursive constructions and discourses in society. Paying close attention to the diversity in the material, I went on to identify explicit and implicit words used in the material line-by-line to construct the discursive object in focus – i.e., hearing voices. When a discursive construction of hearing voices was identified, I started to piece together discursive objects and statements that could be traced to form a coherent logic – i.e., a discourse. I sought to trace and analyze preliminary suggested discourses to broader discourses in society, connecting the micro-level discussions of the material to related macro-level discussions of history, culture, societal institutions, power relations, and ideologies. After identifying a discourse, I could start identifying the subject positions of that discourse within the material, and trace and analyze the limits and possibilities that a given discourse makes available for different subject positions in relation to hearing voices. As with qualitative research in general, the analysis process was cyclical, and I moved back and forth between working on the different criteria. Through both meticulous bottom-up puzzle-piecing and a wide-angled tracing of the identified and named discursive constructions to broader sociocultural connections, the analysis resulted in the identification of six overarching discourses: *biomedical psychiatry-discourse*,* discourse of reason*,* psychodynamic discourse*,* discourse of personal characteristics*,* spiritual discourse and discourse of personal relationships between hearer and voices*.

### Reflections on the research process and the researcher

When my master’s thesis project in psychology commenced in the early 2010s, I was a young, ethnically Norwegian woman without firsthand experiences of voice-hearing. In both my professional and personal life, I sought to position myself as an open-minded non-expert and fellow human being, in solidarity with people who hear voices.

In my experience, literature searches in the 2010s resulted in fewer publications of personal accounts of hearing voices than in 2024. In the 2010s, I also experienced disinterest from editors whom I contacted regarding a discourse analytical inquiry of voice-hearing. However, as the current BMC Psychology special collection on *Mental Health*,* Discourse*,* and Stigma* could be argued to attest to, the past 13 years have brought changes – for instance, in what appears to be a greater interest in discourse analysis in mainstream psychology.

As the instrument for doing this research, I have also changed in terms of continuing to explore, try and fail, and learn as a scholar, as a community psychologist practitioner, and as a fellow human being. To my understanding, the most prominent way in which my combined theoretical, methodological, empirical, practical, and experience-based knowledge (‘the 2024 lenses’) have influenced the inquiry and analysis, compared to the lenses used in the early 2010s, is by widening my perspectives. The widened perspectives have enabled me to be curious about, trace, and discuss more nuances and connections than before, within and between discourses. Nevertheless, the Foucauldian discourse analysis that was conducted in the early 2010s was carefully and thoroughly conducted with the support of my supervisor. The originally identified discourses remain, although one of the discourses has been renamed. This will be pointed out where relevant in the following section, in which the results from the analysis are presented and discussed.

## Results and discussion

### Biomedical psychiatry-discourse: biomedical invalidation of first-person accounts of hearing voices as mere symptoms

The construction of *auditory hallucinations* was among the pronounced ways in which hearing voices was articulated in the young women’s blogging and in the interview with the man. In most of the blogs and the interview, there was a wide range of words and statements interrelated with the *biomedical psychiatry-discourse*, such as auditory hallucinations and being sick, as one would expect to find, given its predominance in relation to matters concerning mental health in many places across the globe [3, 19, 25]. For instance, paraphrasing Nadia, when her auditory hallucinations first began presenting, she thought she was very sick.

The discursive construction of auditory hallucinations was reconstructed by piecing together words and statements from the blogs and the interview – for instance, addressing hallucinations as a mere symptom of serious mental illness, and naming it a symptom of psychosis and schizophrenia. Hallucinations were addressed in the material as needing to be diagnosed, treated, and monitored – preferably by medical doctors and psychologists.

I identified the subject positions of *psychiatric patients*,* medical doctors*, and other *health personnel*. In the material, I found words such as psychotics and schizophrenics, which were analyzed as being a prominent part of the psychiatric patient position. Furthermore, the bloggers and the interview participant explicitly discussed that they had been or were being medicated with antipsychotics, and the bloggers wrote about being in therapy and previous voluntary and forced admissions to institutions.

The following excerpt from the interview with Thomas exemplifies the analysis of the skewed power-relation between the medical doctor and the patient in the biomedical discourse:

Thomas: I’m not so sure about medications having the desired effect on the voices.

Interviewer: No [an ordinary supportive validation to a negation in Norwegian ]. So if I understand… [stopping to avoid talking at the same time].

Thomas: Bu- But um … that’s maybe up to the doctors to decide.

From previous work and certainly from people’s firsthand experiences, it is well-known that questioning and speaking against biomedicine, and sometimes simply speaking about hearing voices, may put the person in a position of risk in encounters in both civil society and the mental health system. This risk may entail real-life psychosocially and physically constraining consequences. The consequences may range from being met with invalidating censorship of first-person accounts, disbelief and ridicule, to forced treatment regimens and forced admissions to psychiatric institutions, as attested to by the bloggers and as documented in the literature [18, 19, 25]. Through discontinuing the questioning and stating that “that’s maybe up to the doctors to decide”, I analyzed that Thomas’ subject position as a patient who hallucinates unavoidably came into play.

Re-analyzing this excerpt 13 years after the first round of analysis, I reconstruct my response, “No. If I understand…”, as something that, despite thorough efforts to position myself as a non-expert in solidarity with people who hear voices, may have made Thomas conscious of my psychologist position and his patient position at this point in the interview. Seen against the backdrop of the predominant biomedical psychiatry-discourse, Thomas’ firsthand knowledge is by default positioned as invalid in relation to making claims about the effect of medications, placing him in a non-dominant, subjugated position that he appeared to enter by placing medical doctors as authorities on the ordering of medication. As discussed elsewhere, the biomedical discourse and its colonization of illness-talk would not be predominant without self-subjugation [25].

However, showing that Thomas knew his subordinate position in the knowledge hierarchy through explicitly acknowledging the authority of the medical profession could be analyzed as also facilitating small windows of opportunity to question the biomedical discourse without risking being fully invalidated as a knower. As Blackman [31] has discussed, after the Second World War, rather than the voices in themselves, another concern grew in terms of how a person relates to their voices, especially whether the person has *insight* or not. As introduced in the Background section, in biomedical psychiatry, to have insight entails viewing one’s voices as empty symptoms of mental illness and, in effect to deny and self-invalidate one’s own experiences of hearing voices. This notwithstanding, the knowledge content of Thomas’ statement resonates with the reality of the young women bloggers and also firsthand accounts from other inquiries in that voices frequently persist despite antipsychotic medication [6, 14].

### Discourse of reason: invalidation of first-person accounts as nonsense

A *sign of madness* was another discursive construction of hearing voices in the analysis, based on explicit descriptions by most of the bloggers and the interview participant as being viewed as mad by themselves or others because of voice-hearing, as in the following quote by Liv:

It’s so embarrassing. Hilda [pseudonym for a voice] keeps giving me wrong answers all the time. It’s so embarrassing to show the teacher the wrong answers. And I can’t tell him that they’re not my answers but her answers. He’ll think that I’m completely mad! Which I am, though, but he doesn’t have to know, does he?!

In analyzing the material, madness appeared to be aligned with *unreason* to a greater extent than a symptom of disease, making the two discursive constructions of hallucinations and madness stand out as qualitatively different, although sharing certain commonalities such as constructing the hearing of someone who is not audible for others as devoid of meaning.

The construction of voice-hearing as madness was found to be localized in a *discourse of reason* by tracing how madness was described in the material. For instance, one may trace several instances of unreason in Liv’s quote, such as the repeated mentioning of the voice Hilda providing wrong answers to Liv, and that these were embarrassing to hand in to the teacher. Further, by sharing the thought of telling the teacher about knowingly submitting wrong answers that had come from a voice that Liv was well aware that other people could not hear, I trace Liv’s articulation as painting an image of what would appear to the teacher to be utter nonsense.

The analysis suggests that a discourse of reason constructs and separates the reasonable from the unreasonable. By tracing explicit and implicit words and statements linked with voice-hearing and unreason in the blog and interview material, such as in Liv’s quote, unreason implicitly stood out as existing in a sphere of incomprehensibility, unpredictability, the imaginary, the potentially destructive, and simply as wrong. In contrast, through analyzing the material, reason stood out as right, being in control, normality, and as being able to determine what is real and not. For instance, in Nadia’s blog, Nadia reflected on standing out as not being normal, but crazy because of the voices. It was written as a quoted statement to her as the recipient (“you are”), together with an image of an item with the words ‘crazy’. In the context it was written in, I read it as alluding to representing the view of general others in civil society when faced with voice hearing. Amalie’s blog provides a different example, of coming to insight: paraphrasing Amalie, she stated to have felt a little bit dumb for not gaining insight sooner into realizing that the voices she heard were not real.

To be clear, I could not trace implicit or explicit articulations in the data material about, for instance, what madness is or what it entails. Madness in terms of unreason did not appear to be articulated with meaning in its own right, other than as nonsense, as the opposite or shadow of reason – through the language of reason. As such, the current analysis of a discourse of reason aligns with the work of Foucault [4] and the discussion that the advent of the Age of Reason came with a silencing of madness, rendering what had been a dialog into a monolog.

I identified the subject positions of *reasonable* and *unreasonable/mad people* in relation to the discourse of reason, as can be traced in the examples from the blogs of Liv, Nadia, and Amalie, where mad people appeared to listen to voices, and reasonable people appeared to know that voices are not real. Through the analysis, reasonable people appeared to be characterized as rational in feeling, thought, and action, and to be worthy of being listened to and respected. Unreasonable persons appeared to be characterized as mad, senseless, abnormal, and potentially dangerous, who may need protection from themselves and should not be taken seriously. The undesirability of being positioned as unreasonable can be traced in Liv’s quote, where she did not disclose to her teacher that voices disturbed her schoolwork to prevent being deemed as mad.

In a discourse of reason – as discussed in relation to Thomas and the biomedical psychiatry discourse, and drawing on Blackman [31] – insight appears as a central signifier of whether the person is capable of the degree of self-control that is considered necessary for taking responsibility for their wellbeing and actions. As analyzed and discussed in a previous project concerning community mental health care, there appear to be connections between the discourse of reason and the discourse of liberalism in relation to who is afforded autonomy and who is viewed by others and themselves as needing to be more closely socially regulated and cared for [25]. This differentiated affordance of autonomy, depending on the considered capacity for self-regulation, also seems to resonate with discussions in the service users’ movements, especially from contexts where the discourse of liberalism holds a strong position, such as North America [25]. The discussion related to autonomy will be returned to and further developed in relation to the discourse of personal characteristics, in a later sub-section.

Revisiting the analysis 13 years after the first round, it may be considered in light of the recently established field of Mad Studies (e.g., 3). For instance, studies and discussions about sanism facilitate questioning and problematizing the utterly derogatory, stigmatizing, and invalidating positioning of the mad person in a discourse of reason. As attested to by the term ‘Mad Studies’, engaging with critique and widened perspectives appears to be paving the way to reclaim *Mad* as a hard-earned title to be proud of, similar to the reappropriation of the title *Queer* by the LGBTQI+-movement (cf. Mad pride) [3]. If the blogs and the interview had been from 2024, perhaps the discourse of sanism could have been traced in the material. However, in the current analysis words and statements connected with madness were traced solely to the discourse of reason. Following Gayatri Spivak’s [32] postcolonial analysis of the subaltern as someone without a validating language of their own, the unreasonable or mad person, as positioned in a discourse of reason, indeed appears to fit that description with an understanding of madness as utter *nonsense*. As exemplified by the following quote by Thomas, this analysis suggests that the discourse of reason continues the historical silencing of persons who hear voices if they do not want to be deemed as mad and potentially dangerous in encounters in civil society: “But at least I act normally in the community,” referring to his voices talking while other people were around, without him talking back.

The decision to not talk about voices may be analyzed as engaging in an act of self-censorship, as discussed by Semino, Collins, and Demjén [23] and by myself and colleagues elsewhere [25]. Semino, Collins, and Demjén [23] analyzed actively not talking about hearing voices with other people as a major form of silence in the experience of voice-hearing. It was discussed as highly demanding for a person to tell others about experiences of hearing voices in general. Even merely talking about voices was viewed as private and, at the same time, as something that was experienced as upsetting for loved ones to hear about. On the whole, the authors [23] analyzed and discussed hearing voices as *untellabl*e, even in intimate, trusting relationships.

### Psychodynamic discourse: paternalizing firsthand accounts

Another discursive construction I identified in some of the blogs was that voices and visions were depicted as being a symbolic expression of a person’s needs, thoughts, feelings, and traumas, and as such, voices appeared as having a *symbolic function*. For instance, as Liv discussed in the following quote, her psychotherapist’s view on her voices was that they were a result of-, and had the function of symbolizing-, the hidden meanings of repressed traumatic experiences and, especially, sexual abuse during her upbringing.

Then Alexander [pseudonym for a voice] became the topic in terms of what they [therapists] believe that the voices really are. But I do not agree. They believe that Alexander and the others [voices] have come about from my repressed thoughts and traumas that I have experienced in the past. That is, a type of voice-hearing that follows dissociative disorder.

… I don’t know what they [voices] are or where they come from, but they are real. If I could only prove it, but I can’t.

Through the analysis, as a clinically trained psychologist, I located voice-hearing as a symbolic function in a *psychodynamic discourse*, demarcating life stories and persons in need of therapy and therapists from those not in need. I identified the subject positions of the *patient who hears voices* and the *therapist* in a psychodynamic discourse. The patient was characterized as someone who is considered as not knowing what the voice-hearing and sometimes visions symbolize, as may be traced in Liv’s quote. Liv stated that she disagreed with her therapist’s interpretation that her voice-hearing merely symbolizes traumatic experiences in her life, such as sexual abuse. However, since Liv could not disprove it to her therapist, she had to live with the final say being the expert opinion. Being enabled to articulate resistance against a discourse, as Liv did in this passage, often entails the embedded presence of other discourses at work. The two other discourses that one may see slight traces of in this quote are the upcoming discourse of spirituality and personal relationships.

In contrast, the analysis delineates a subject position for the therapist as the required expert in interpreting the symbolism that the voices bear. This consequently entails allowing and requiring the voice-hearing patients to pay attention to and talk about their experiences of voice-hearing. Voice hearers can disagree with their therapists’ interpretations, as shown in Liv’s quote. However, as the decision-making party, therapists have the final say regarding the most meaningful interpretations.

Others may analyze the discursive objects and subject positions identified in this material as drawing on other trauma-related discourses. For instance, HVM-pioneer Romme and colleagues [6] consider voices in relation to the lived life of the given person, without necessitating a psy-professional theoretical lens, as more recent inquiries have also done – for instance, that by McCarthy-Jones et al. [19]. In relation to lived life-perspectives, hearing voices is often discussed in connection with people’s personal ways of coping and recovery processes following, for instance, traumatic hardships and struggles in life. In her firsthand account of hearing voices, Longden [18] shared that in her recovery process, she came to view her voices as normal reactions to such hardships in life.

Nevertheless, based on the blog material analyzed here, as exemplified by Liv’s quote above, and with my clinical and theoretical lenses and knowledge of the Norwegian mental health system, the psychodynamic discourse stood out as salient. For example, I traced explicit and implicit talk about hearing voices explained as repressed and dissociated thoughts, feelings, and memories in need of release and that are transformed and projected into something experienced as external existences. Furthermore, as mentioned in Liv’s quote, therapy concerned with such discursive objects was discussed as something that goes on in the DPS. In the context of Norway, community-based psychiatric centers are called *distrikspsykiatriske sentre* (DPS). These are welfare-state-provided regional centers for mental health care, such as in-patient- and out-patient therapeutic facilities. Access to patient rights and nationally recommended treatments are considered and granted on the basis of psychiatric diagnosis and psychosocial distress. Psychodynamic treatments are among the standard therapies that are available and listed in the Norwegian Clinical Practice Guidelines for psychotic diseases, with the most prominent treatment ordained being antipsychotics [33].

As a professional clinical system of knowledge, the psychodynamic discourse shares commonalities with the biomedical discourse – for instance, in terms of objectives of clinically solving problems and privileging the views of professionals. However, articulating voice-hearing as something symbolically meaningful signifies a clear break with biomedical discourse and discourse of reason, and their total silence and monolog, instead facilitating dialog.

Spivak’s [32] version of the postcolonial concept of the subaltern also comes into play here. In the biomedical discourse and the discourse of reason, the person hearing voices is so othered that whatever is said about voice-hearing is rendered as nonsense. In contrast, the words uttered about hearing voices in the psychodynamic discourse are considered as possibly making sense. However, the way to decipher voice-hearing as sensemaking is through the paternalizing caveat of having to be analyzed and interpreted by an expert analyst. Thus, the firsthand knowledge of the person hearing voices is still, in effect, invalidated by only being considered as sensemaking after it has been filtered through professional logic. Persons hearing voices as such remain subaltern through their words and experiences being filtered through professional discourse. This filtering is similar to what a previous project has analyzed in relation to the discourse of human development related to rules against talking about illness in Norwegian community-based meeting places. Also there, we analyzed a similar doubleness of well-meaning professionals and strides being made towards “facilitated horizons of possibility” and being constrained by professionals being considered as knowing best [34, p.70].

### Discourse of personal characteristics: validation or responsibilisation?

The analysis of some of the blog material and the interview also reconstructed voice-hearing as *a personal characteristic* of people who hear voices. As an example, I paraphrase from Nadia’s blog that referred to statistics showing that a majority of those who hear voices are able to manage their voices on their own and do not need psychiatric help. She furthermore referred and linked to online publications where professionals with ties to the HVM were interviewed or were the authors (e.g., 35), for instance informing that only a minority of persons who hear voices meet the criteria for diagnosis related to psychosis and schizophrenia. As such, hearing voices appeared to be discussed as a kind of personal characteristic rather than solely a psychiatric symptom.

I located this construction of voice-hearing within a *discourse of personal characteristics*, constructing and separating naturally varying characteristics from psychiatric symptoms in a continuous, rather than discrete fashion. An example can be traced in this paraphrase from Sofie’s blog where she stated that she believed that voice-hearing is something that varies naturally in the population, similar to other characteristics. Sofie wrote about being interested in natural sciences, so I read her discussion of hearing voices as naturally varying as alluding to evolutionary theory and natural selection. Hearing voices as a personal characteristic aligns with a fundamental understanding of the HVM of voice-hearing as *a human experience*, rather than pathology in itself [12, 36]. As addressed in the Background section, viewing voice-hearing as a human experience resonates with research that attests to there being between 4 and 10% of the general population who report hearing voices – for most of whom it is not related to diagnosable distress (e.g., 7, 8, 9).

I have identified the subject positions within a discourse of personal characteristics as *voice-hearers* and *professionals in support of the HVM*. As mentioned in relation to paraphrasing Nadia, professionals in support of the HVM were portrayed as advocating for human rights and societal acceptance of voice-hearers, and critiquing biomedical psychiatry’s predominance. In the analysis of the material, voice-hearers were implicitly positioned to have the prerogative to discuss and manage their own voice-hearing. However, the emphasis on the separation between a personal characteristic and a psychiatric symptom, as illustrated in the paraphrasing of Nadia, may suggest that being in control over one’s voice hearing could also be key in this discourse.

The analysis suggests that voice-hearing is rendered a potential problem without self-regulation as in the three previous discourses. The separation between hearing voices as a personal characteristic and as a psychiatric symptom could imply that greater autonomy depends on expectations to self-monitor and adequately regulate one’s voice-hearing, especially when voice-hearing starts to signify a risk – as previously discussed by Blackman [31]. As such, the professional control over voice-hearing associated with the previous three discourses here appears to be shifting to the governing of the self. Walkerdine [37] has analyzed and discussed the disciplined governing of the self as being rendered the pinnacle of individual development of late modernity. The governmentality concept *technologies of the self* stands out as relevant, as it conceptualizes governing by positioning the person to constantly monitor, control, and improve themself [38]. This shift of responsibility may involve an often subtle process that has been termed responsibilization in the governmentality literature. *Responsibilization* refers to the process wherby something that was previously considered the responsibility of, for instance, the welfare state and professionals, trickles down to increasingly becoming viewed and accepted as the private responsibility of the individual [39]. Together with colleagues, I analyzed the presence of responsibilization in relation to the topic of service user involvement in Norwegian community mental health care in a previous inquiry [40]. This has also been analyzed and discussed by Terkelsen [33] in relation to antipsychotic treatment regimes in Norway.

### Spiritual discourse: articulating spiritual engagement with voices

Hearing voices that others cannot hear was also reconstructed from some of the blog material as *personal contact with the spiritual and supernatural*. For instance, in some posts from Liv’s blog, she discussed that her voices and visions may belong to spirits of another realm. The discursive construction of personal contact with the spiritual and supernatural was located in a *spiritual discourse*. Historically, in European countries such as Norway and the United Kingdom, the spiritual discourse and, more specifically, the Christian discourse were in predominance concerning communication with spirits until the Age of Enlightenment [19]. Although Norway upholds a state-funded *Christian folk church* and traditions, Norway could be described as a country where secularity and religious plurality are typically valued [41]. Thus, it was unsurprising that the way in which the spiritual was described in the blog material was less religious and more connected with spirituality and the supernatural.

In terms of the analysis of subject positions, compared to the four previous discourses, the spiritual discourse entails a clear difference, whereby the voice being heard is not just an object to be experienced but also represents *spirits and supernatural beings*, with their own mystical and autonomous subject positions. I also identified the subject position of persons hearing voices as *persons who can sense and communicate with spirits*, also known as mediums. An example may be found in Sofie’s blog, where she stated that sometimes when she was alone in her flat, she saw a being resembling a person there.

Following the material and analysis, spirits and supernatural beings were analyzed as wanting something from persons who can communicate with them, as illustrated in the following quote from Liv’s blog: “He [Alexander] says that if I obey him, he will take me with him to another, forbidden world where everything is amazingly beautiful, quiet and calm. There I am free.”.

Furthermore, spirits/supernatural beings were positioned as possessing special powers, such as the ability to travel to a forbidden world as seen in the quote by Liv. Persons who were able to interact with spirits and supernatural beings were depicted as possessing supernatural abilities. Although spirits were described as more powerful than the persons who can communicate with them, the spirits were positioned as dependent on the medium in order to impose themselves on material reality. Furthermore, the exorcising of spirits/supernatural beings was depicted as feasible for persons who can communicate with them, as illustrated in Sofie’s blog, where she told of having successfully removed the being in her flat with a spiritual séance-ritual.

Historically, in Christianity, some of those who claimed to be communicating with God were validated and positioned as prophets, and later as saints [19]. Even in a secularized country such as Norway, some persons have gained a somewhat validated position as supernaturally gifted in the general public opinion, such as the now deceased *Snåsamannen* (the man from Snåsa) – who was considered to be able to heal and have psychic powers (e.g., 42). However, there seem to be few public contexts in which people not already positioned as renowned clairvoyants may talk about voice-hearing and where their accounts would be taken seriously. However, in the HVM and among firsthand knowers of hearing voices, one of the pronounced ways of accounting for voice-hearing is as a spiritual experience [12, 27, 36].

### Discourse of personal relationships between hearer and voices: articulating everyday life with voices

Lastly, I have reconstructed the hearing of voices as *personal communication and interaction between hearer and voice*, based on material from most of the blogs and the interview. Despite similarities with the spiritual discourse, this discursive construction of hearing voices was described as resembling interactions with ordinary people rather than being related to the supernatural sphere. This separation may be viewed in light of Norway’s positioning as a secularized country.

The analysis points to situating the discursive construction of personal communication and interaction between hearer and voice within a *discourse of personal relationships between hearer and voices.* In the first round of analysis in the early 2010s, I chose a more technical and abstract way of naming this discourse as a *discourse of interactive co-existence between hearer and voices*. However, I consider that *discourse of personal relationships* is a name that better articulates everyday life with voices in a more intuitively understandable, everyday language on the basis of feedback I have received over the years when teaching psychology students about discourse analysis, my own continuous learning process, and a growing amount of research literature addressing relational aspects of hearing voices [e.g., 20, 21, 43].

In the analysis of subject positions within this discourse, both *voice-hearers* and *voices* were positioned as subjects, as in the spiritual discourse. Voices were portrayed as separate but conjoined with the hearer and as sounding like the speech of tangible persons. As may be traced in the following quote from Liv’s blog, voices could have names and recognizable characteristics:

As early as grade school, Alexander came around, in a kind of sneaky manner, as a longed-for friend, conversation partner, father, uncle, brother – a grown up that I could trust. He talked – I answered. I talked – he answered. Back and forth. Nothing mysterious, nothing scary, only a good friend that no one else could hear. Only mine!

Voice hearers and voices were articulated as relating to each other through personal interactions and delineated relationships in everyday life, as illustrated in Liv’s quote. In the analysis, voices were portrayed with a variety of ways of being; sometimes a given voice was discussed as complex and having both positive and constructive and negative and destructive sides, or as more one-sided, as increasingly addressed in the literature and by the HVM [6, 12, 18–21, 36].

In the analysis, voices were sometimes described as engaging in monologs – for instance, in Liv’s blog, she told about different messages she had been given from the voice Alexander, such as being told to defend herself when she was bullied in school as a child, or to cut herself as a young woman. As exemplified in Liv’s quote above, voices were also described as engaging in dialogs. Voices and the person hearing them were also discussed as influencing each other’s experiences and actions. For instance, Thomas stated: “When a person is happy, then the voices make you grumpy; if you’re grumpy, then the voices … they try to make you happy.”

The analysis suggests that a discourse of personal relationships between hearer and voice stands out as a set of statements describing the personal and even private life of a person, as seen from the unique vantage point of a firsthand knower of particular relationships concerning everyday living with voices—the know-how of hearing voices. The diversity of ways of experiencing personal relationships with voices subsumed in this discourse resonates with the expected and necessary messy heterogeneity of people’s everyday life encounters and relationships. As discussed by Parker [44], in order to promote social justice for people in psychosocial hardships, a bare minimum of necessary action is to respect and validate firsthand experiences of psychosocial differences in their diversity and complexity. This discourse tells of ongoing, daily interactions and relationships between hearers and voices that appear to be a part of life, even with the use of antipsychotic medications, as indicated by the bloggers and the participant interviewed in this inquiry.

### Conclusion and closing reflections

With the emergence of biomedical psychiatry in the Age of Enlightenment, dialogs about madness were silenced. Through the silencing, persons who hear voices were arguably left without validating sensemaking languages for their firsthand experiences. In the current inquiry, I have been in search of languages and knowledge that may facilitate sensemaking of voice-hearing for persons who hear voices. I aim to contribute to validating firsthand knowledge by partaking in the building of a critical mass of research-based knowledge about firsthand accounts of psychosocial differences and hardships, that may actively promote change. I have engaged in Foucauldian discourse analysis to analyze five Norwegian firsthand accounts of hearing voices, which comprised four sets of blogs authored by four young women, and a short interview with a man. Furthermore, I analyzed how identified discourses diverged from the predominating biomedical psychiatry discourse. Six discourses were identified: *biomedical psychiatry-discourse*, *discourse of reason*, *psychodynamic discourse*, *discourse of personal characteristics*,* spiritual discourse*,* and discourse of personal relationships between hearer and voices.*

The analysis suggests that within the discourses of biomedical psychiatry and reason, voice-hearing appeared as hallucinations, unreason, and as a problem to be solved. In line with Foucault’s historical analysis, the current analysis supports that the biomedical psychiatry-discourse and the discourse of reason silence the person who hears voices, with consequences of exacerbating hardships rather than alleviating them (e.g., 3, 18). Here, the proper response to voices not going away is the use of antipsychotic medications and encouraging voice hearers to ignore and deny them [31, 33].

In contrast, the analysis suggests that within the discourses of psychodynamics, personal characteristics, spirituality, and personal relationships between hearer and voices, voice-hearing appears to be portrayed as something that can be made sense of by the firsthand knower. However, the analysis also shows that the psychodynamic discourse aligns with the discourses of reason and biomedical psychiatry by allowing professionals the prerogative of determining the meaning of voice-hearing and thereby retaining their professional power [13].

In somewhat different ways, the discourses of personal characteristics, spirituality, and personal relationships between hearer and voice appear to facilitate languages that allow greater personal freedom and more space for persons who hear voices to reflect on, narrate, and generally make meaning of their voice-hearing at their own discretion, with little to no professional impingement being needed.

The discourse of personal characteristics appears to resonate with the HVM [12]. The HVM argues for privileging voice-hearers’ own interpretations concerning their voice-hearing rather than the opinions of professionals, thereby contributing to validating firsthand knowledge and representing an alternative to the predominating discourses concerning voice-hearing. However, the current analysis also suggests that lack of control over one’s voice-hearing could become a problem. In the analysis of the discourse of personal characteristics, lack of control over one’s voice-hearing appeared to be discussed as possibly needing to be dealt with by psy-professionals. This may position this discourse as potentially entangled with more professionally driven discourses. However, in contrast to the emphasis placed on professional clinical knowledge in the traditional mental health system (to which, for instance, biomedicine and psychodynamics have been connected in the analysis), the HVM emphasizes more holistic and recovery-oriented approaches to distressing voices with the lived life of a person as the starting ground. The HVM promotes approaches to distress that respect and validate the ways of seeing and dealing with voices that make sense for the person, or that may make sense for the person after gaining familiarity with different ways of knowing voice-hearing [13]. In line with the HVM and the wider service users’ movement, the help offered to those in need should, at the very least, be experienced as *helpful help* by those on the receiving end, and service-user participation should of course saturate the collaboration between the person and the helper concerning the personal recovery process.

The spiritual discourse and the discourse of personal relationships depart from the four previous discourses by articulating voice-hearing as relational and entailing encounters between two subject positions rather than between one subject and an object. In analyzing these discourses, one gets glimpses of unique insider knowledge about everyday living with and engaging with voices. This know-how of hearing voices is only attainable for people who do not hear voices by carefully listening to the firsthand knower. Here, the tables have turned from the silenced psychiatric patient to the person who hears voices being positioned as the expert – the expert by experience – while the professional without personal experiences is rendered the less knowledgeable party in need of being taught by the firsthand knower.

To ask whether a spiritual discourse and a discourse of personal relationships entail facilitated horizons of possibility for the person hearing voices is not straightforward and would require a more thorough discussion of the concept of empowerment than is possible here. Considering solely the practical real-world dimension of everyday living with voices, as with other personal relationships, it is clear that relationships with voices could be destructive and constructive (e.g., 18, 20). This notwithstanding, in everyday living with voices, simply having languages available that enable a person to narrate their own experiences and actions at their own discretion fosters a more independent position. Such languages may empower and in a manner free firsthand knowers to make meaning of and validate everyday life on their own without having to depend on a professional to pass judgment on and to validate whether their way of making sense of their life makes sense or not, which – resonates with the Mad Pride movement’s slogan “The right to be free, the right to be me”.

In the final paragraphs of this article, I engage in a brief methodological discussion. The aim of the present article was to identify and analyze firsthand accounts of hearing voices and how they differed from the predominating biomedical psychiatry-discourse in search of validating languages and knowledge that may facilitate sensemaking of voice-hearing for people who hear voices. Foucauldian discourse analysis facilitates explorations of voice-hearing without attributing it to *essences inside* persons and rather links it to available discourses and thus the social and cultural conditions that have made the experiences and subject positions possible, as well as to the investigation of potentials for change [28]. Within this framework, predominant discourses and their naturalized objects are also exposed as discursive constructions, and other, potentially more emancipating and empowering discourses can be illuminated as better alternatives.

The six discourses that I have identified and analyzed are considered to be products of the particular data material of this inquiry, and of the particular analyst who generated the original analysis in the early 2010s, that is me – with the support of my master’s thesis supervisor. They are also the products of the same analyst, with 13 years of gathered experiences and knowledge, who revisited the analysis and discussion in 2024, and are set in the context of the social democratic welfare state of Norway during two periods in time. I adhere to the social constructionist understanding that research is created in the interactions between those who are being researched and the researcher, and as such making the researcher’s influences on the research an inseparable part of the inquiry. Thus, changing any of the ingredients involved with a particular research project and analysis, such as using material from blogs from 2024, could conceivably have led to a different discourse analysis with different results and discussions. I have striven to describe the inquiry and the analysis undertaken in a transparent and accountable manner. Discourse analysis is not intended to make generalizations; rather, I have sought to make the generated knowledge available so that the reader may draw on it theoretically to illuminate and discuss different ways of seeing and being with voices in other settings, at the reader’s discretion.

A limitation of the inquiry is that, in order to protect the anonymity and integrity of the bloggers, I did not directly quote three of the four blogs in the article – considering the ease of translating back to Norwegian with AI engines and searching the web for the original blogs. For future blog-based inquiries, I suggest inviting authors of firsthand accounts who freely consent to openly collaborate with the inquiry and to openly share their accounts for research purposes. Another limitation is that the discourse analysis was conducted without firsthand knowledge. To continue working towards validating firsthand languages and knowledge about hearing voices for persons who hear voices, for future inquiries, I suggest engaging in participatory discourse analysis (e.g., 34) together with persons who have firsthand knowledge of voice-hearing from different walks of life.

## Data Availability

The datasets generated and/or analyzed during the current study are not publicly available of concern for the anonymity and integrity of participants but are available from the corresponding author on reasonable request.
